# Global Research in Aging

**DOI:** 10.1093/geroni/igaf122.257

**Published:** 2025-12-31

## Abstract

In this session research is presented from across the globe to address important geriatric syndromes.

